# Combined photodynamic therapy and intravitreal bevacizumab as treatment for nonresponsive myopic choroidal neovascularization

**DOI:** 10.4103/0974-620X.71911

**Published:** 2010

**Authors:** Jay Kumar Chhablani

**Affiliations:** Department of Ophthalmology, Shiley Eye Center, MC 0946, 9415, Campus Point Drive, Rm 217B, La Jolla, CA 92093, California

**Figure 1:**
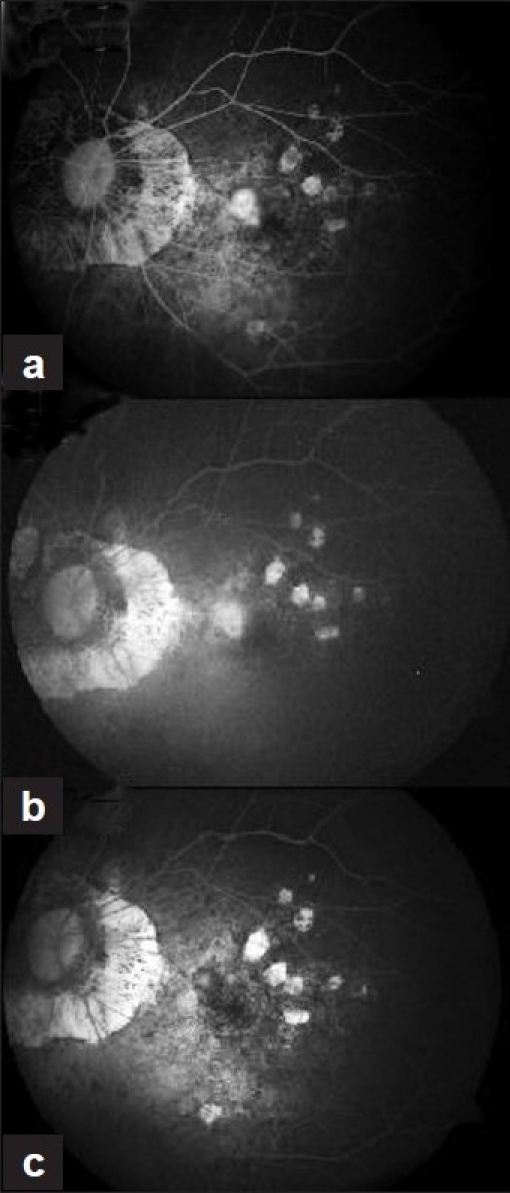
Serial Fundus Fluorescein Angiography (FFA) showing decrease in leakage at the area of choroidal neovascular membrane

**Figure 2:**
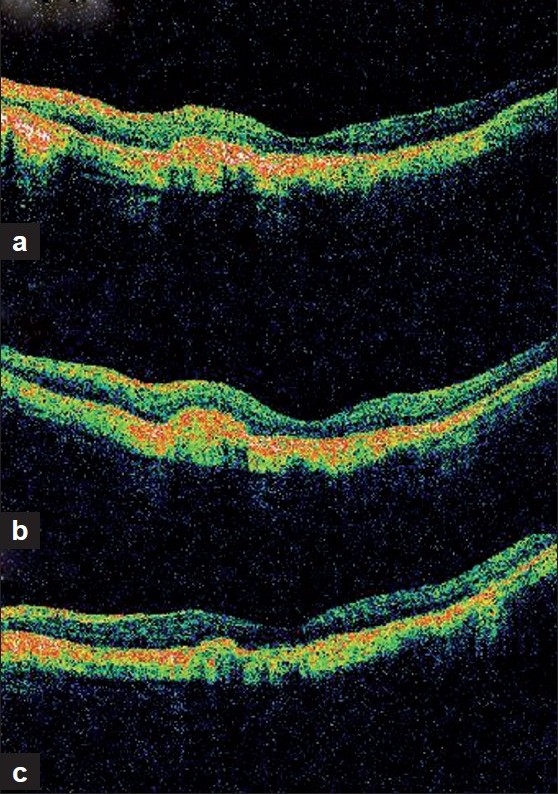
Serial Optical Coherence Tomography (OCT) showing decrease in retinal thickness with restoration of normal foveal contour

Sir,

We report the efficacy of combination therapy using photodynamic therapy (PDT) and intravitreal bevacizumab for resistant choroidal neovascular membrane (CNVM) associated with myopia. A 59-year-old man presented with decreased vision in the left eye since 10 days. He did not have any systemic complaints. On examination, his best-corrected visual acuity was 6/18 in the right eye and 6/12 in the left eye. Anterior segment examination of both eyes was unremarkable, but for pseudophakia. Intraocular pressure in both the eyes was 15 mmHg. Fundus examination in right eye showed a myopic disc, rest of the fundus was within normal limits. Fundus examination in left eye showed myopic disc with subretinal hemorrhage. Fundus Fluorescein Angiography (FFA) [Figure 1a] and Optical Coherence Tomography (OCT) [Figure 2a] of the left eye confirmed the presence of juxtafoveal CNVM. The patient underwent PDT as per standard protocol and intravitreal injection of triamcinolone 2 days later.

At 1-month follow-up visit, his best-corrected visual acuity was decreased to 6/24 in left eye with the presence of persistent activity on FFA [Figure 1b] and retinal thickening on OCT [Figure 2b]. Combination therapy using PDT with anti-vascular endothelial growth factor (VEGF) was considered. Patient underwent PDT followed by intravitreal bevacizumab (1.25 mg) injection, 2 days later. No treatment-related adverse effect was noted.

At the 1-month follow-up visit, his best-corrected visual acuity had improved to 6/9 in left eye, but FFA showed persistent activity. A second injection of intravitreal bevacizumab was performed.

At the 2-month follow-up visit, his visual acuity was maintained at 6/9 with no activity on FFA. He has been on regular follow-up with stable fundus findings. At the 1-year follow-up visit, his visual acuity was maintained at 6/9 with scarred CNVM clinically and no activity on FFA [Figure 1c] with normal foveal contour on OCT [Figure 2c]. Patient was advised home Amsler’s monitoring and review after 4 months.

Combination therapy plays a synergistic effect in the treatment of choroidal neovascularization. The combined regime is postulated to be beneficial in reducing the need for cyclic injections.[1] VEGF inhibition alone can prevent neovascularization at an early stage. However, once neovascular beds are established, they are unlikely to regress with anti-VEGF therapy alone. At this stage, a combined approach using a non-thermal laser has been seen to be beneficial.[2] Our patient did not respond to intravitreal triamcinolone with PDT, but showed significant improvement after subsequent administration of two injections of intravitreal bevacizumab along with PDT. Intravitreal bevacizumab alone has been reported to show better results compared to PDT.[3] Combination therapy with PDT could be considered as a better alternative to intravitreal bevacizumab alone, to reduce the number of injections and maintain the success for the long term, specially in resistant cases.
